# Exploring the Metatranscriptome of Bacterial Communities of Two Moss Species Thriving in Different Environments—Terrestrial and Aquatic

**DOI:** 10.3390/plants13091210

**Published:** 2024-04-26

**Authors:** Vesselin Baev, Gana Gecheva, Elena Apostolova, Mariyana Gozmanova, Galina Yahubyan

**Affiliations:** 1 Department of Molecular Biology, Faculty of Biology, University of Plovdiv, Tzar Assen 24, 4000 Plovdiv, Bulgaria; eapostolova@uni-plovdiv.bg (E.A.); mariank@uni-plovdiv.bg (M.G.);; 2 Department of Ecology and Environmental Conservation, Faculty of Biology, University of Plovdiv, Tzar Assen 24, 4000 Plovdiv, Bulgaria; ggecheva@uni-plovdiv.bg

**Keywords:** Hypnales, *Hypnum cupressiforme*, *Platyhypnidium riparioides*, metatranscriptome, sequencing, host microbiome, bacterial communities, moss

## Abstract

Mosses host diverse bacterial communities essential for their fitness, nutrient acquisition, stress tolerance, and pathogen defense. Understanding the microbiome’s taxonomic composition is the first step, but unraveling their functional capabilities is crucial for grasping their ecological significance. Metagenomics characterizes microbial communities by composition, while metatranscriptomics explores gene expression, providing insights into microbiome functionality beyond the structure. Here, we present for the first time a metatranscriptomic study of two moss species, *Hypnum cupressiforme* (Hedw.) and *Platyhypnidium riparioides* (Hedw.) Dixon., renowned as key biomonitors of atmospheric and water pollution. Our investigation extends beyond taxonomic profiling and offers a profound exploration of moss bacterial communities. Pseudomonadota and Actinobacteria are the dominant bacterial phyla in both moss species, but their proportions differ. In *H. cupressiforme*, Actinobacteria make up 62.45% and Pseudomonadota 32.48%, while in *P. riparioides*, Actinobacteria account for only 25.67% and Pseudomonadota 69.08%. This phylum-level contrast is reflected in genus-level differences. Our study also shows the expression of most genes related to nitrogen cycling across both microbiomes. Additionally, functional annotation highlights disparities in pathway prevalence, including carbon dioxide fixation, photosynthesis, and fatty acid biosynthesis, among others. These findings hint at potential metabolic distinctions between microbial communities associated with different moss species, influenced by their specific genotypes and habitats. The integration of metatranscriptomic data holds promise for enhancing our understanding of bryophyte–microbe partnerships, opening avenues for novel applications in conservation, bioremediation, and sustainable agriculture.

## 1. Introduction

Mutually beneficial interactions between microorganisms and plants contribute essential nutrients and substrates necessary for microbial proliferation, thereby directly or indirectly enhancing plant growth and overall health [1]. The bacteria affiliated with host organisms primarily encompass both endophytes residing within the hosts and epiphytes residing on the external surfaces. Consequently, the intricate host niche is shaped by the colonized tissues and organs, the plant’s genotype, and the environmental conditions, collectively influencing the bacterial community associated with the plant host.

Bryophytes (mosses, liverworts, and hornworts), as non-vascular plants, demonstrate a capacity to acclimate to highly fluctuating environmental conditions. Most moss species exhibit a noteworthy resilience to desiccation, promptly restoring normal metabolic activities upon rehydration. Their unique role in ecosystems is supported by interactions with bacteria, possibly impacting mineral nutrition, carbon economy, herbivory, and growth and development [2]. Bryophyte assemblages are usually developed in places unsuitable for other plant species but for microorganisms. Bryophytes are associated with diverse microbiota, including bacteria, fungi, and cyanobacteria. The microbiota of bryophytes plays a crucial role in their survival and growth, and the interactions between bryophytes and their associated microorganisms are complex and diverse [3,4]. Bacterial communities associated with bryophytes are dominated by the phyla Proteobacteria and Actinobacteria, with large amounts of unclassified bacteria also present [5]. Nevertheless, few studies have been conducted on these microbial communities’ nature and bio-functional diversity, and understanding their coexistence is especially interesting [6].

Recent studies have also demonstrated the extensive application of the moss-bag technique in the biomonitoring of airborne particulate matter [7], trace elements [8], and heavy metals [9] associated with air pollution. Aquatic mosses have also an undisputable role in water quality assessment, confirmed over many years of research [10]. The technique involves the utilization of mosses to accumulate specific substances or elements during the monitoring processes. Due to their adaptive features, mosses emerge as proficient hosts for the establishment of symbiotic microbial communities [11]. Monitoring environmental pollution requires not just using mosses but also understanding the importance of the bacteria they host. These bacteria play a critical role in key biological processes, such as breaking down harmful substances, especially when mosses are used to collect specific elements [12,13,14]. Therefore, it is imperative to gain a comprehensive understanding of moss-associated bacteria. Recently, it has been revealed that mosses even employ chemical signaling mechanisms to attract bacteria, which in turn contribute to shaping their microbiome [15,16,17]. Consequently, mosses establish intricate connections within a diverse microbial network, endowing them with enhanced capabilities to procure nutrients, fend off pathogens, and exhibit resilience in the face of environmental stressors such as limited water availability, extreme temperatures, and UV radiation [3,18,19].

Moss microbiomes exhibit a fundamental assembly of bacteria consistently found in numerous prevalent boreal and tundra moss species [20,21]. While the moss microbiome typically demonstrates a strong host specificity [21,22], host identity alone does not exclusively dictate microbiome composition and can be heavily influenced by local environmental conditions [23,24]. The connections between bacteria and bryophytes have historically not received enough attention [25]. However, a handful of recent researchers have commenced exploring intriguing roles that bacteria might assume in the physiology of bryophytes. These roles may include, but are not limited to, moss defense [26], nitrogen fixation, the control of moss growth and development stages [27], CO_2_ production, anoxygenic phototrophy [28], and freeze protection [29], among others.

To comprehend the implications of climate change on the involvement of mosses in ecosystem nitrogen cycling, it is imperative to investigate the response of moss-associated microbial communities to ecosystem-wide warming. Variations in environmental factors and, thus, in microbiota composition may consequently influence N2-fixation rates [30], possibly through physiological adjustments of diazotrophic communities [31]. Numerous investigations have concentrated on the nitrogen fixation associated with prevalent moss species, namely *Pleurozium schreberi* (Willd. ex Brid.) Mitt., *Hylocomium splendens* (Hedw.) Schimp., *Ptilium crista-castrensis* (Hedw.) De Not., and various Sphagnum species. The substantial contribution of these moss species to environmental nitrogen levels, particularly in boreal forests and to a moderate extent in tundra ecosystems, was demonstrated by Rousk et al. [32], Carella and Schornack [33], and Jean et al. [34]. The nitrogen fixation of different mosses is primarily attributed to the activity of proteobacteria or cyanobacteria. Additionally, several moss species harbor a broader, although currently unidentified, range of N-fixing bacteria. These bacteria likely compete or occupy distinct niches, contributing to complementary roles in diazotrophic processes within the moss environment [28].

Tang and coworkers [35] analyzed the bacterial community linked to ten liverwort and ten moss host species in Tibet, China. The consistently prevalent bacteria belonged to the phyla Acidobacteriota, Actinomycetota, Armatimonadota, Bacteroidota, Planctomycetota, and Pseudomonadota. Ma and coworkers [36] also observed that Proteobacteria and *Actinobacteria* were the two most abundant phyla in several analyzed mosses. Examination of metagenome datasets of bog ecosystems commonly dominated by Sphagnum mosses reveals the prevailing bacterial constituents, categorized into six phyla: Proteobacteria, Acidobacteria, Actinobacteria, Verrucomicrobia, Bacteroidetes, and Chlamydiae [5].

Opelt and Berg [13] conducted an identification study, revealing numerous antagonistic bacteria linked to three moss species (*Tortula ruralis*, *Aulacomnium palustre*, and *Sphagnum rubellum*). These bacteria belonged to nine distinct genera, with Burkholderia, Pseudomonas, and Serratia emerging as the dominant ones. Another investigation delved into the functional roles and diversity of bacterial species associated with two Sphagnum species (*S. fallax* and *S. magellanicum*) thriving in a temperate mire ecosystem. Species within the Burkholderia genus were prevalent in association with both Sphagnum species, suggesting their potential involvement in pathogen defense, and nitrogen fixation processes [23,37]. Tian et al. [38], reported that in *Hygroamblystegium noterophilum*, *Entodon compressus*, and *Grimmia montana,* the most dominant phyla were Proteobacteria, Bacteroidetes, Actinobacteria, and Acidobacteria. Saha et al. [39] examined bacteria linked to the moss *Plagiomnium rostratum*. Their findings revealed that the prevailing bacterial species belonged to the families Bacillaceae (Bacillota), Enterobacteriaceae (Pseudomonadota), Lactobacillaceae (Bacillota), Pseudomonadaceae (Pseudomonadota), and Moraxellaceae (Pseudomonadota).

To gain a more profound understanding of how a microbial community works, scientists are increasingly adopting extensive metatranscriptomics approaches involving RNA-seq, which can depict the actual functional landscape of the bacterial community. However, the metatranscriptomics reports of the moss bacterial community remain very limited. This study presents, for the first time, the bacterial communities of two moss species from the Order Hypnales, namely *Hypnum cupressiforme* Hedw. and *Platyhypnidium riparioides* (Hedw.) Dixon. Our goal was to clarify both the taxonomic and functional profiles of the associated microbiota archiving it by applying RNA-seq metatranscriptomics. Furthermore, we explored the structure and composition of the nitrogen cycle gene (sub)families in microbiomes of the two moss species. The findings of this investigation contribute valuable insights into the intricate interplay between bacteria and mosses, thereby enhancing our understanding of this complex ecological system.

## 2. Materials and Methods

### 2.1. Sample Collection and Processing

**Figure 1:**
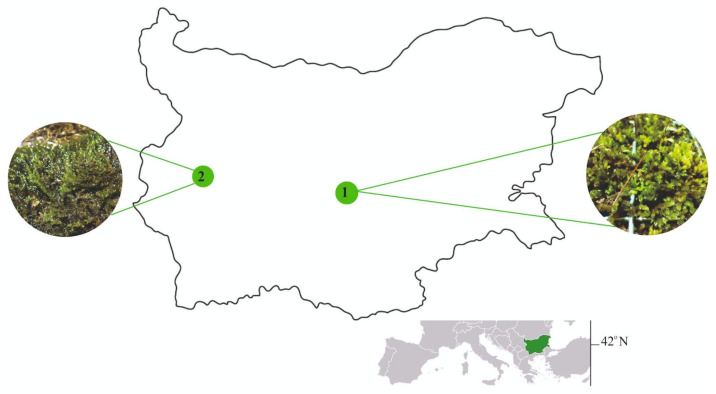
Localities where moss samples were collected in the territory of Bulgaria: 1—*H. cupressiforme*, 2—*P. riparioides*.

*H. cupressiforme* was collected from Sredna Gora Mountain, Central Bulgaria; 42°28′06.0″ N 24°56′07.9″ E, altitude: 600 m a.s.l. (Figure 1). Moss material was sampled from rocks within the oak forest openings in February 2023 in a region representative of non-urban areas. *P. riparioides* was sampled from Dragalevska River, Vitosha Mountain, SW Bulgaria, near the capital Sofia; 42°37′26.0″ N 23°18′19″ E, altitude: 872 m a.s.l. Aquatic moss was collected from stones in August 2023 along the river near the Vitosha Nature Park. 

### 2.2. Total RNA Extraction and Sequencing

The total RNA was extracted using the RNeasy Plant Mini Kit (Qiagen, Valencia, CA, USA). RNA quality and quantity were checked with a Qubit 4 Fluorometer (Invitrogen™, Thermo Fisher Scientific, Waltham, MA, USA). Samples with an RNA integrity number (RIN) ≥ 7 determined by the Qubit RNA IQ assay were used for further analysis. The total RNA was sequenced by Illumina PE150 (Illumina NovaSeq; San Diego, CA, USA), with rRNA depletion (Novogene Co., Ltd., Cambridge, UK). The two libraries were with 22 M and 27 M reads in *H. cupressiforme* and *P. riparioides*, respectively. The raw FASTQ files were used for the downstream analysis. The sequencing data were submitted in GenBank under the BioProject accession number PRJNA1093277.

### 2.3. Bioinformatics Analysis

The paired-end sequence reads in FASTQ format, which underwent filtration and adapter removal, were analyzed using Kraken2 against the PlusPFP database [40,41]. This database encompasses entries for archaea, bacteria, viruses, plasmids, humans, UniVec_core, protozoa, fungi, and plants. The analysis was conducted with reference to the specified database available at https://benlangmead.github.io/aws-indexes/k2 (accessed on 23 April 2024). The samples were decontaminated from plant reads and remaining reads attributed to bacteria were used for further analysis and re-classification with standard full Kraken2 DB. Results of the Kraken2 were analyzed and visualized using the Pavian program (https://fbreitwieser.shinyapps.io/pavian/, accessed on 23 April 2024). For the downstream analysis and functional profile, we used the WGSA2 workflow with default parameters [42]. KEGG database was used for the functional analysis [43], and for the visualization AmpVis2 were used [44].

To analyze nitrogen-cycling genes using shotgun metatranscriptome sequencing data, we employed the manually curated functional gene database NCycDB1 [45]. In summary, raw read sequences were queried against NCycDB databases through DIAMOND (v 2.1.9). Sequences matched with NCycDB were extracted to generate functional gene profiles for nitrogen-cycling microbial communities. The Perl script within NCycDB facilitated the extraction of functional profiles. A random subsampling procedure was implemented to normalize the total number of sequences for each sample to the minimum sequencing depth [45].

## 3. Results and Discussion

### 3.1. Community Structure Analysis of Bacteria Associated with Moss Hosts

The reads obtained from RNA sequencing underwent decontamination to eliminate plant-related sequences. In *H. cupressiforme*, 72.86% of the reads were classified as Viridiplantae. Similarly, in *P. riparioides*, 77.03% were identified as Viridiplantae. The remaining reads were subjected to downstream microbial analysis.

Recently, Groß et al. [46] indicated that non-cyanobacterial diazotrophs might prevail in temperate mosses, consistent with earlier research on boreal mosses conducted by other groups [3,21,35,36]. Their findings revealed that Cyanobacteria constituted very few of the bacterial communities, with Pseudomonadota (Proteobacteria) and Actynomicetota (Actinobacteria) emerging as the most abundant bacterial phyla in boreal moss species. Proteobacteria encompass organisms with both photoautotrophic and chemolithotrophic capabilities, with certain members possessing the capacity for nitrogen fixation and forming symbiotic associations with bryophytes [21]. Wang et al. [47] also explored microbiomes of different moss species through 16s rRNA and showed that they mainly consisted of Proteobacteria and Actinobacteria.

**Figure 2:**
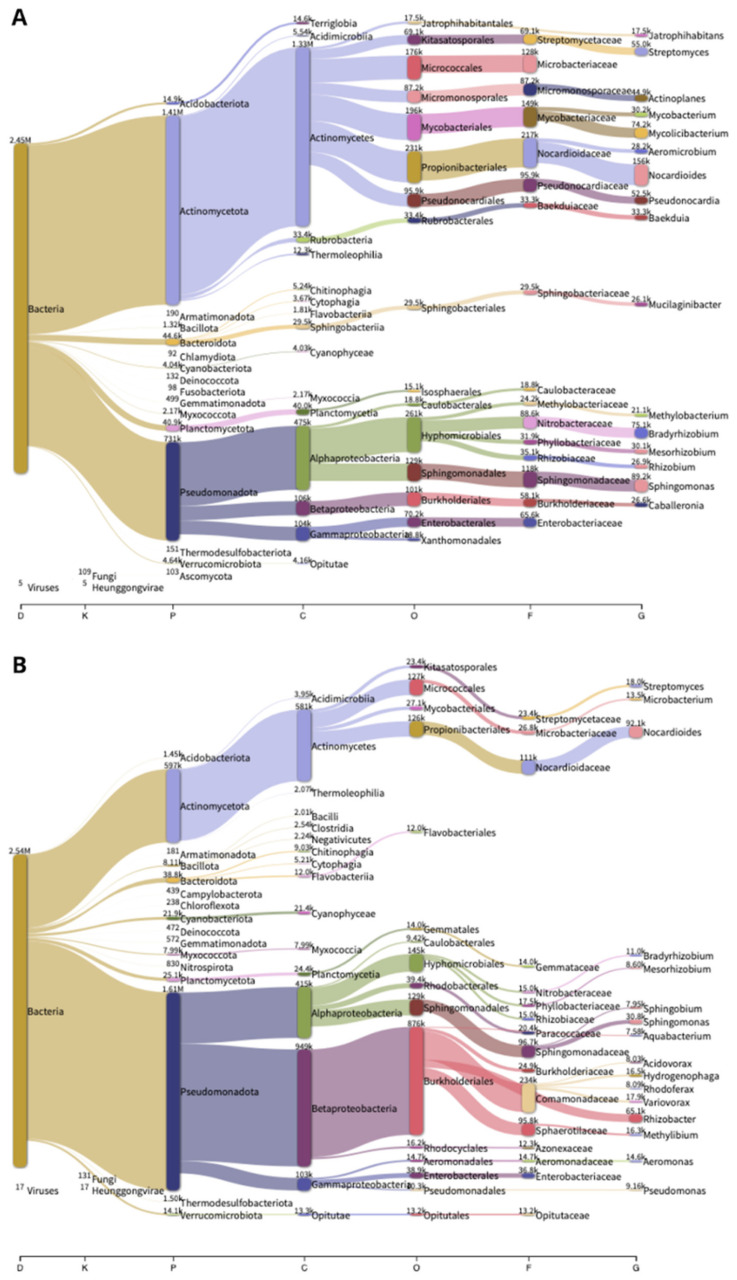
Sankey diagram displaying the composition of microbiota at all taxa levels in the two mosses. (**A**) *H. cupressiforme* and (**B**) *P. riparioides.* The colored columns from left to right represent the proportions of bacterial taxa from domain to genus levels.

At the phylum level, our analysis yielded consistent results for both samples, with Pseudomonadota and Actinobacteria dominating, collectively comprising more than 95% of the bacterial communities of the two moss species. Notably, there was a notable difference in the prevalence of these two phyla within the microbiomes of the two moss species. In *H. cupressiforme*, Actinobacteria constituted 62.45%, while Pseudomonadota accounted for 32.48% (Figure 2A). Conversely, in *P. riparioides*, Actinobacteria comprised only 25.67%, whereas Pseudomonadota dominated with 69.08%, as depicted in Figure 2B.

The ecological implications of these differences in phyla abundance can be of significance. For example, the relative abundance of Pseudomonadota and Actinobacteria in the Arabidopsis holobiont can influence the plant’s response to biotic and abiotic stresses, as these bacterial phyla are known to play important roles in plant growth promotion, biocontrol, and nutrient cycling. Similarly, the abundance of Actinobacteria in the endophytic compartment of A. thaliana can affect the plant’s resistance to pathogens, as Actinobacteria are known to produce a variety of bioactive compounds with antimicrobial properties [48,49]. In the rhizosphere of plants grown in Chilean extreme environments, the abundance of Proteobacteria can influence the plant’s ability to survive in harsh environments, as these bacteria are known to play important roles in nitrogen fixation, phosphate solubilization, and other processes that are critical for plant growth in nutrient-poor soils [50]. Since certain bacteria may be a defense against abiotic stress, it would be of practical benefit to compare the microbiomes of the biomonitor *H. cupressiforme* at different levels of atmospheric pollution.

Additionally, less prominent phyla in bacterial communities of the two analysed moss species included Planctomycetota, Bacteroidota, Acidobacteriota, Cyanobacteriota, Bacilliota, Myxococcota, and Verrucomicrobiota (Figure 2). Growth trials revealed that Planctomycetes dwelling in peat can break down numerous heteropolysaccharides found in Sphagnum peat. The introduction of Sphagnum peat increased the proportional abundance of Planctomycetes compared to the overall microbial community [51,52,53,54]. In *H. cupressiforme* microbiom, the relative abundance of Planctomycetes was higher (1.81%) compared to *P. riparioides* (1.07%). The Bacteroidota exhibited a moderate presence in moss-dominated environments and also in our samples. Previous studies have identified this phylum in conjunction with moss phyllidia, suggesting they may offer some level of protection to mosses against freezing [29,55].

On the genus level, the most abundant bacteria from Pseudomonadota were *Sphingomonas* (8.16%), *Bradyrhizobium* (6.87%), *Mesorhizobium* (2.75%), *Rhizobium* (2.46%), *Methylobacterium* (1.93%), and *Caballeronia* (2.44%), and from Actinomycetota, they were *Nocardioides* (14.31%), *Mycolicibacterium* (6.79%), *Streptomyces* (5.04%), *Pseudonocardia* (4.81%), *Actinoplanes* (4.11%), *Baekduia* (3.05%), *Mycobacterium* (2.77%), and *Aeromicrobium* (2.58%) in *H. cupressiforme*. On the other hand, in *P. riparioides*, the most abundant Pseudomonadota genera were *Sphingomonas* (4.65%), *Bradyrhizobium* (1.66%), *Rhizobacter* (9.83%), *Variovorax* (2.70%), *Hydrogenophaga* (2.48%), *Methylibium* (2.46%), *Aeromonas* (2.21%), and from Actinomicetes, there were *Nocardioides* (13.91%), *Streptomyces* (2.71%), and *Microbacterium* (2.04%). Detailed information on all common and specific genera in both samples can be found in Supplementary Table S2.

The most abundant genus from Proteobacteria, *Sphingomonas*, in *H. cupressiforme* was previously connected with nitrogen fixation [56] and with the production of growth-promoting substances [38]. *Rhizobacter*, the dominant genus in *P. riparioides*, the generalist from Proteobacteria, is among the nitrogen fixers [57]. 

In both host species, *Nocardioides* were the most abundant of the actinomycetes. *Nocardioides* is likely to have a remarkable impact on biological weathering. The rhizosphere of mosses contains abundant actinomycete communities, including *Nocardioides*, which are recognized as bacteria involved in rock weathering due to their ability to extend hyphae deep into the rock and release organic acids that dissolve rock composites [58].

The two samples not only vary in the proportion of Pseudomonadota and Actinobacteria but also exhibit differences in the composition of genera within each group. In *H. cupressiforme*, the dominant Actinobacteria phylum exhibits a higher abundance at the genus level compared to Pseudomonadota, reveiling host-specific variations in genera such as *Pseudonocardia*, *Actinoplanes*, *Baekduia*, *Mycobacterium*, and *Aeromicrobium*. Conversely, in *P. riparioides*, the prevailing Pseudomonadota phylum is characterized by host-specific abundance in genera such as *Rhizobacter*, *Variovorax*, *Hydrogenophaga*, *Methylibium*, and *Aeromonas*, among others.

In *H. cupressiforme*, one of the most prevalent genera is *Bradyrhizobium*, comprising 6.87%, whereas in *P. riparioides*, it constitutes only 1.66%. Similarly, discrepancies are observed in the genus *Rhizobium*, with 2.46% in *H. cupressiforme* compared to 0.53% in *P. riparioides*. Conversely, the genus Rhizobacter dominates in *P. riparioides*, accounting for 9.82%, while it represents only 0.08% of the microbial composition in *H. cupressiforme*. Certainly, the variations observed in the microbiome as host-specific are likely attributed to the intricate interactions between the individual hosts and their unique living environments.

### 3.2. Functional Analysis of the Microbiome of the Two Moss Hosts

Metagenomic analysis has proven highly effective in characterizing microbial communities, primarily focusing on their composition. Another limitation of metagenomics is that the DNA pool extracted from various moss samples typically contains nucleic acids originating from living, dormant, and dead microbes [38,59]. In contrast, metatranscriptomic analysis provides a valuable complement by exploring gene expression patterns and regulatory mechanisms. This dual approach significantly enriches our comprehension of microbiome functional dynamics, offering insights beyond mere community structure. Most previous studies have relied on 16s or shotgun metagenomics, leaving a gap in exploring the functional aspects within the bacterial community and its interaction with the host, which can be addressed through metatranscriptomics [3,6,18,19,21,24,27,35]. To our knowledge, only a few publications have utilized metatranscriptomics as a method to unveil novel aspects of microbial communities associated with the well-studied peat moss Sphagnum [60,61].

By absorbing atmospheric CO_2_ and participating in the nutrient cycling process, photosynthetic microbes act as elemental reservoirs within the bryosphere. They contribute nutrients to plants [15,62], serve as a food source for animals [63], and support other microorganisms [64,65]. Additionally, photosynthetic microbes are likely to influence carbon flux exchange between the bryosphere and the atmosphere [66].

**Figure 3:**
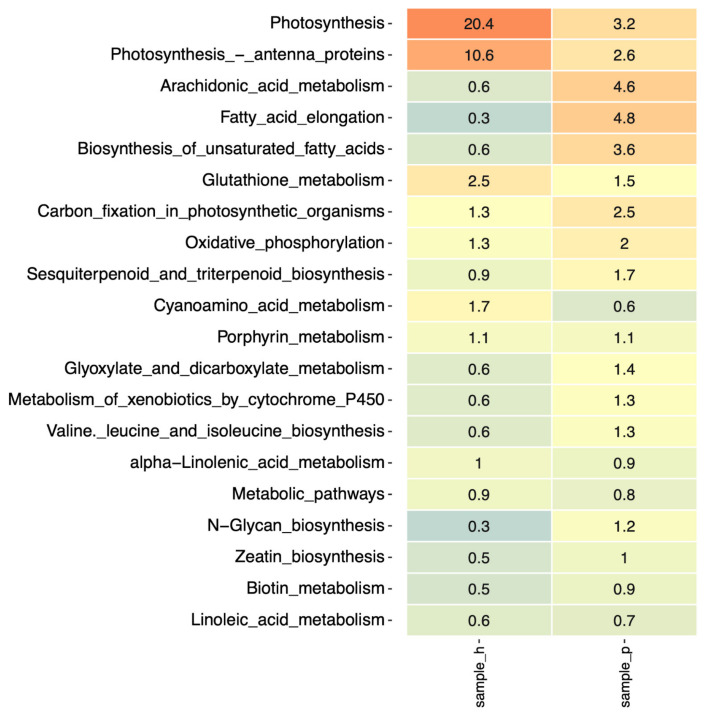
Heatmap of relative abundance of the top 20 metabolic pathways in the bacterial communities of the two in *H. cupressiforme* (sample_h) and *P. riparioides* (sample_p).

Our data show that the microbial community of *H. cupressiforme* expresses more transcripts related to photosynthesis pathways than *P. riparioides* (Figure 3). This outcome may be explained by both different host species that predetermined different bacterial communities and also different environmental factors of the host location. Cyanobacteria and other photosynthetic bacteria, for example, within the genus Bradyrhizobium, may be more active within the specific bacterial community regarding these functional pathways.

The different abundance in the functional pathways may also be defined by the host and microbiome interaction established via diverse metabolic interconnections between them. The variety of bacterial symbiotic relationships accessible to mosses is believed to be diminished compared to earlier stages of their evolutionary development. This reduction is attributed to the substantial loss of genes facilitating intracellular, mutualistic symbioses in the majority of moss genomes, as outlined by Radhakrishnan et al. [67]. Despite this, contemporary mosses engage in diverse ecological associations with bacteria. These interactions encompass mutually beneficial exchanges of resources and, alternatively, antagonistic relationships where bacteria disrupt and potentially manipulate the physiological functions of the host, as discussed by Carella and Schornack [33].

Unlike *H. cupressiforme*, *P. riparioides* metabiome is enriched in several lipid metabolism pathways (Figure 3). Our data show that in the *P. riparioides* microbiome, the fatty acid pathways are more abundantly presented than in *H. cupressiforme*. The biosynthesis of unsaturated fatty acids in bacteria plays a crucial role in their adaptation to various environmental conditions. Bacteria have developed mechanisms to regulate fatty acid metabolism, including the synthesis of unsaturated fatty acids, to adapt to changes in their surroundings. While the pathway for unsaturated fatty acid synthesis is not widely distributed in bacteria, certain species possess mechanisms for producing these essential fatty acids [68]. Studies have shown that bacteria can modulate the levels of unsaturated fatty acids to respond to external stimuli and environmental cues, highlighting the importance of fatty acid metabolism in bacterial adaptation [69]. The higher abundance of acid pathways in the *P. riparioides* microbiome should be considered in light of its aquatic environment—more pronounced temperature and oxygen fluctuations, physical stressors like strong currents. Fatty acids, in particular, could contribute to temperature adaptation, gas exchange, and absorption of nutrients and could reduce mechanical stress.

While bacteria do not typically contain arachidonic acid in their membranes, they may encounter this fatty acid in their environment or during interactions with plant hosts. In some cases, bacteria have developed mechanisms to metabolize or modify fatty acids present in their surroundings. Nevertheless, bacteria can produce arachidonic acid as seen in a study where an Antarctic bacterium was found to contain elevated proportions of the fatty acids [70]. The biosynthesis of polyunsaturated fatty acids like arachidonic acid by bacteria is considered a selective adaptation to temperature and environmental conditions. Moreover, arachidonic acid and its derivatives are pivotal in plant stress signaling networks, impacting the plant’s ability to resist insects and pathogens [71]. This suggests that these metabolite pathways could contribute to the interaction between the host and bacterial community and vice versa.

In the symbiotic relationship between plants and bacteria, glutathione plays a critical role in mediating interactions and ensuring symbiotic success. Research indicates that the bacterial glutathione pool is essential for the growth of bacteria during interactions with plant partners, affecting nitrogen fixation capacities [72]. Mutant strains with altered glutathione levels exhibit oxidative stress and impaired symbiotic abilities, highlighting the importance of glutathione in bacterial symbiosis with plants [72]. Moreover, glutathione is crucial in preventing early senescence and abnormal nodule development in bacteria like *Rhizobium tropici*, emphasizing its significance in maintaining symbiotic relationships [73,74]. Our data showed higher activity of genes related to glutathione metabolism in the microbiome of *H. cupressiforme* (Figure 3) Moreover, glutathione in bacterial cells serves various essential functions, including maintaining redox balance, defending against oxidative stress, modulating virulence gene expression, aiding detoxification processes, and contributing to optimal biofilm formation and adaptation to different environments [73,74].

An early aspect in examining bryophyte interactions with bacteria was the notion that bacteria serve as a CO_2_ source for bryophytes, especially in aquatic environments Wetzel et al. [75]. We have observed that the carbon fixation pathway is more abundant in the microbiome of *P. riparioides*, which prefers fast-flowing waters, but may occur sparsely in ditches, canals, and ponds.

Despite lacking specialized structures like nodules found in certain plants, mosses have been recognized for their potential contribution to nitrogen fixation processes by harboring nitrogen-fixing bacteria. These bacteria establish symbiotic relationships with mosses, residing in specialized structures known as mucilage pockets or cavities on the moss surfaces.

Nitrogen-fixing bacteria in moss–microbe associations convert atmospheric nitrogen into ammonia, enhancing nitrogen availability for plant uptake. Mosses play a crucial role in nitrogen cycling, particularly in nutrient-poor environments like boreal and tundra ecosystems. Research continues to unveil the diverse mechanisms by which mosses contribute to nitrogen cycling and ecosystem nitrogen dynamics [76], acting as primary supplier of nitrogen in some ecosystems [32]. Over the preceding decades, considerable endeavors have been directed toward delineating nitrogen cycle pathways across diverse ecosystems, employing various methodologies. Recently, metagenome and metatranscriptome sequencing has emerged as a valuable tool to investigate gene families associated with the nitrogen cycle, linking them to environmental processes [77,78,79].

In terms of functionality, the microbial functional traits that mediate nitrogen-cycling processes exhibited a relatively uniform and consistent distribution across various moss species. Here, applying the NCycDB, we were able to detect the expression of 59 genes in the *H. cupressiforme* microbiome and 67 genes in the *P. riparioides* microbiome, respectively (Table 1 and Supplementary Table S1). The nitrification genes amoABC_A (archaea) were not detected in samples, whereas the amoABC_B bacterial genes from this pathway were mostly detected in the *P. riparioides* microbiome, suggesting that the bacteria play a role in ammonia oxidation in this host. Another difference between the two microbiomes concerns particulate methane monooxygenase (pmoABC) genes, only presenting in *P. riparioides*, which suggests the potential role of aquatic mosses in methane oxidation. Both samples lack the anammox genes responsible for hydrazine production from ammonia and nitric oxide (hzo, hzsA, hzsB, hzsC, hdh).

Furthermore, numerous research endeavors directed towards understanding N2-fixing microbiomes have concentrated on sequencing and identifying the nifH gene in plants [80,81,82,83]. To identify the potential bacteria that contribute to nitrogen fixation, we have explored the reads mapped to nitrogen fixation genes nifDHK (nifW gene was not detected in samples) (Supplementary Table S1). It is crucial to highlight that, given the metatranscriptomic nature of the data, these findings indicate not merely the existence of the genes within bacterial genomes but rather the active gene expression, mirroring the functional involvement of the bacterial community in the respective process.

Our results indicate a reliance on Actinomycetia for nitrogen fixation within the microbiomes of both moss species, with a notably higher prevalence in *H. cupressiforme*. Specifically, the nitrogen-fixing microbiome predominantly hosts Alphaproteobacteria, whereas *P. riparioides* exhibits a higher presence of Betaproteobacteria. These data are also coherent with the total taxonomic distribution of the two host species. Moreover, the microbiome of *H. cupressiforme* revealed the expression of nif genes from various genera, including *Sphingomonas, Bradyrhizobium*, *Mesorhizobium*, *Burkholderia*, *Sphingobium*, *Caballeronia*, *Rhizobium*, and *Nitrobacter*, whereas *P. riparioides* showed a different landscape of genera—*Roseateles*, *Sphingomonas*, *Sphingopyxis*, *Rhizobacter*, *Rhodobacter*, *Rubrivivax*, *Erythrobacter*, *Bradyrhizobium*, *Burkholderia*, *Hydrogenophaga*, and *Pseudomonas*. Bradyrhizobium has attracted extensive attention in research, primarily because it encompasses species capable of nodulation-associated diazotrophy, forming symbiotic relationships with plants [84,85]. *Bradyrhizobium*, *Rhizobium* and *Mesorhizobium*, collectively known as rhizobia, were more abundant in the terrestrial *H. cupressiforme* microbial community.

These microbial associations contribute to essential functions such as nutrient cycling, stressor resistance, and overall host health. Moreover, they play a crucial role in defending the moss host against potential pathogens, contributing to its resilience and survival. Understanding the intricate dynamics of the microbiome functional role within moss hosts not only sheds light on the intricate web of ecological interactions but also highlights potential applications in ecosystem management, conservation, and even biotechnological advancements.

## 4. Conclusions

Mosses harbor diverse bacterial communities that are crucial for nutrient acquisition, stress tolerance, and pathogen defense, existing in intricate symbiotic relationships. Understanding their functional capabilities is vital for grasping their ecological significance fully. We present the first metatranscriptomic study of two moss species, *Hypnum cupressiforme*, and *Platyhypnidium riparioides*, exploring their bacterial communities beyond taxonomic profiling. Our analysis revealed Pseudomonadota and Actinobacteria as the dominant phyla, with evident differences in their relative abundance between the two moss species. We suggest that the abundance and composition of bacterial phyla in the moss-associated microbiota can vary significantly depending on the host species, the environment, and the specific compartment of the plant (e.g., endophytic or epiphytic) and can influence the mosses’ ability to thrive in the specific environments. Further research is needed to fully understand the ecological and functional roles of these bacterial communities in plant growth, development, and health. We detected most genes involved in nitrogen cycling and observed variations in pathways like CO_2_ fixation, photosynthesis, and fatty acid biosynthesis, suggesting potential metabolic discrepancies between the two moss species. The metatranscriptomics dual approach of integrating expression patterns and regulatory mechanisms may further enrich our understanding of bryophyte–microbe partnerships, enabling novel applications in conservation, bioremediation, and sustainable agriculture. Moss-associated bacterial communities can serve as early indicators of ecosystem disturbance, notably in conservation and climate change contexts. Studying these communities aids in safeguarding biodiversity, crucial for ecosystem balance and climate change mitigation. Additional forthcoming investigations can include exploring various moss species across diverse environmental conditions. For example, regarding the water management and bioremediation perspective, it would be valuable to explore the microbiomes of host species that survive eutrophication, e.g., *Leptodictyum riparium* (Hedw.) Warnst. In addition to metatranscriptomics, further studies may rely on an interdisciplinary approach, including proteomics and metabolomics, which can further elucidate the intricate interconnections between the microbiota and its host species, leading to a deeper understanding and unveiling new insights.

## Figures and Tables

**Table 1:** Nitrogen cycling genes detected in the two metatranscriptomes.

Biological Process	Expressed NCyc Genes
Nitrification	***amoA_B*, *amoB_B*, *amoC_B*, *hao*, *nxrB***
Denitrification	*napA*, *napB*, *napC*, *narG*, *narH*, *narJ*, *narI*, *nirK*, *nirS*, *norB*, *norC*, *nosZ*, *narZ*, *narY*, *narV*, *narW*
Assimilatory nitrate reduction	*nasA*, *nasB*, *nirA*, *NR*, *narB*, *narC*
Dissimilatory nitrate reduction	*napA*, *napB*, *napC*, *narG*, *narH*, *narJ*, *narI*, *narZ*, *narY*, *narV*, *narW*, *nirB*, *nirD*, *nrfA*, *nrfB*, *nrfC*, *nrfD*
Nitrogen fixation	*anfG*, *nifD*, *nifH*, ***nifK***
Organic degradation and synthesis	*ureA*, *ureB*, *ureC*, *nao*, *nmo*, *gdh_K00260*, *gdh_K00261*, *gdh_K00262*, *gdh_K15371*, *gs_K00264*, *gs_K00265*, *gs_K00266*, *gs_K00284*, *glsA*, *glnA*, *asnB*, *ansB*
Others	*hcp*, ***pmoA*, *pmoB*, *pmoC***

Genes in bold are genes only detected in *P. riparioides*; in underline are genes only detected in *H. cupressiforme*.

## Data Availability

The sequencing data were submitted in GenBank under the BioProject accession number PRJNA1093277.

## Supplementary Materials

The following supporting information can be downloaded at: https://www.mdpi.com/article/10.3390/plants13091210/s1, Table S1: Nitrogen cycling genes detected in the two metatranscriptomes; Table S2: Genus-level taxa identified in *H. cupressiforme* and *P. riparioides* bacterial communities.
